# DAILY EMOTION REGULATION AND AFFECT ACROSS WORK AND FAMILY CONTEXTS IN MID LIFE

**DOI:** 10.1093/geroni/igad104.1404

**Published:** 2023-12-21

**Authors:** Madeline Nichols, Kelly Chandler

**Affiliations:** Oregon State University, Corvallis, Oregon, United States; Oregon State University, Corvallis, Oregon, United States

## Abstract

Emotion regulation is an important skill for adults to use in interpersonal situations; however, limited research has examined whether emotion regulation strategies vary by context or the potential influences these strategies have on daily affect. We examined the frequency of emotion regulation strategies used in work and family contexts and the extent to which these strategies predicted daily positive and negative affect (PA; NA). Middle-aged adults (N=27, Mage=60.15, SD=3.77; Female=91.67%) completed 7 daily diary surveys. In both contexts, adults most often reported modifying situations to be more positive and using situational approaches. At work, adults also frequently expressed their emotions; at home, they reported modifying situations to be less negative. Preliminary multilevel model results indicated that NA was significantly higher on days when individuals reported distracting themselves from work situations (Est.=7.36, p<.01), suppressing emotions at work (Est.=3.29, p<.01), or expressing emotions around family (Est.=4.41, p<.01) compared to days when they did not use these strategies; conversely, on days when individuals distracted themselves from family situations, they reported lower NA (Est. =-2.80, p=.03) than on days they did not. On average, people who modify family situations to be more positive report higher PA (Est.=6.73, p<.01) than those who do not. Our results suggest that there is daily variability in emotion regulation strategy use and consequential impacts on affectual wellbeing by context. Understanding which emotion regulation strategies yield the most benefit in different contexts may inform interventions targeting emotion regulation as a modifiable behavior to promote health and wellbeing in later life.

